# Childbirth complications in Brazil: An analysis of socioeconomic, epidemiological, and health care factors

**DOI:** 10.1590/1980-549720260053

**Published:** 2026-10-19

**Authors:** Narayani Martins Rocha, Wanessa da Silva de Almeida, Célia Landmann Szwarcwald

**Affiliations:** I Fundação Oswaldo Cruz, Institute of Communication and Scientific and Technological Information in Health – Rio de Janeiro (RJ), Brazil.; II Fundação Oswaldo Cruz, Evandro Chagas National Institute of Infectious Diseases – Rio de Janeiro (RJ), Brazil.

**Keywords:** Parturition, Natural Childbirth, Cesarean section, Obstetric labor complications, Unified Health System, Brazil

## Abstract

**Objective:**

To analyze the factors associated with the occurrence of childbirth complications in Brazil.

**Methods:**

A cross-sectional study was conducted using data from the 2019 National Health Survey (*Pesquisa Nacional de Saúde* – PNS). Data from 2,875 women were analyzed. Chi-square tests and logistic regression were used to investigate the association between sociodemographic characteristics, childbirth care indicators, and the occurrence of childbirth complications (outcome).

**Results:**

The prevalence of childbirth complications was 12.3% (95%CI 10.6–14.2). Childbirth complications were more prevalent among women with lower educational attainment (14.4%) and lower income (13.9%), chronic hypertension (37.1%) or hypertension occurring only during pregnancy (29.8%), cesarean delivery (13.9%), and delivery through the Brazilian Unified Health System (*Sistema Único de Saúde* – SUS) (14.5%), compared with their respective reference groups (p<0.05). The multivariate logistic regression model showed significant associations between the outcome and chronic hypertension (OR=5.49; 95%CI 2.90–10.41), gestational hypertension (OR=4.18; 95%CI 2.47–7.08), absence of blood pressure measurement during prenatal visits (OR=2.16; 95%CI 1.01–4.67), delivery through SUS (OR=1.99; 95%CI 1.06–3.71), cesarean delivery without labor (OR=1.75; 95%CI 1.10–2.80), and medically indicated cesarean delivery (OR=1.90; 95%CI 1.06–3.41). Among women who experienced childbirth complications, the odds of neonatal problems were higher (OR=4.12; 95%CI 2.48–6.85), as were the odds of low birth weight (OR=2.59; 95%CI 1.58–4.23) and preterm birth (OR=2.43; 95%CI 1.60–3.68).

**Conclusion:**

Clinical factors and characteristics of childbirth care were associated with the occurrence of childbirth complications in Brazil. Furthermore, women who experienced childbirth complications had higher odds of adverse neonatal outcomes.

## INTRODUCTION

Childbirth is a significant event in the reproductive cycle and an important indicator of the quality of maternal and child health care. Despite advances in the coverage of and access to health services in recent decades, weaknesses in obstetric care persist, as reflected in the occurrence of preventable maternal and neonatal complications^1-4^.

Regional and socioeconomic inequalities exacerbate this scenario. Low-income women residing in the North and Northeast regions face greater difficulty accessing quality services, making them more vulnerable to delays in care, the search for an available hospital bed, and deliveries under precarious conditions^5,6^. These disparities directly affect maternal and neonatal morbidity and mortality^7^, which remain above international sustainable development targets.

Although public policies such as the *Rede Cegonha* (Stork Network) and the National Humanization Policy have sought to humanize care and improve the quality of childbirth care by reducing unnecessary interventions^8-10^, institutional resistance and structural and human resource barriers limit the implementation of these guidelines^11^.

The core of this issue lies in maternal complications occurring during childbirth or the postpartum period, which remain the leading causes of maternal mortality in the country and often result from delays in diagnosing and appropriately managing complications^12^. The occurrence of these adverse outcomes is exacerbated by the indiscriminate use of interventions, such as oxytocin administration, episiotomy, and unnecessary cesarean sections, which increase short- and long-term obstetric risks^13,14^.

From a neonatal perspective, problems affecting the infant may be related to the conditions of childbirth and the type of care received. The most common complications result from delays in fetal extraction or inadequate monitoring of fetal well-being^15^. Prematurity, often associated with elective cesarean sections performed before 39 weeks of gestation, increases the newborn's vulnerability to infections and respiratory complications^16^. This situation may be exacerbated by a lack of infrastructure and trained professionals for neonatal resuscitation, contributing to preventable adverse events^17,18^.

In light of the above, this study aimed to analyze factors associated with the occurrence of childbirth complications using data from the 2019 National Health Survey (*Pesquisa Nacional de Saúde* – PNS). The factors considered included sociodemographic characteristics, indicators of the adequacy of prenatal and childbirth care, the presence of arterial hypertension, and their associations with maternal and perinatal problems.

## METHODS

### Data source

This study used data from the 2019 edition of PNS, specifically Module S (Prenatal and Childbirth Care), which collects information on prenatal care and childbirth care for the most recent birth.

The PNS is a nationwide household survey conducted by the Brazilian Institute of Geography and Statistics (*Instituto Brasileiro de Geografia e Estatística* – IBGE) in partnership with the Ministry of Health. It is part of the IBGE Integrated System of Household Surveys (*Sistema Integrado de Pesquisas Domiciliares* – SIPD). Its primary objective is to generate representative data on the health profile of the Brazilian population, including health conditions and risk factors, and to inform the assessment of health system performance^19^.

The 2019 PNS was approved by the National Research Ethics Commission (*Comissão Nacional de Ética em Pesquisa* – CONEP) in August 2019 (Opinion No. 3.529.376).

### Sampling

The PNS sample was selected as a subsample of the IBGE Master Sample using a three-stage sampling process. In the first stage, Primary Sampling Units (PSUs) were selected by simple random sampling, taking into account the stratification of the Master Sample. In the second stage, households within each PSU were selected by simple random sampling. In the third stage, one household resident was randomly selected to answer the individual questionnaire^20^.

The sample consisted of women aged 15 years or older who had given birth at least once in the two years preceding the survey, that is, between July 28, 2017, and July 27, 2019.

### Study variables

- Outcome: Experienced a childbirth complication, measured by the question, "Did you experience any complication during childbirth?", as self-reported by the participants.
- Sociodemographic variables
  a) Maternal age group, obtained from the question "Age of the resident on the reference date" and subsequently categorized as 15–24 years, 25–34 years, and 35 years or older;
  b) Educational attainment, obtained from the question "Highest level of education attained," with categories grouped as incomplete high school, incomplete higher education, and completed higher education;
  c) Per capita household income, derived from the question "Per capita household income range" and categorized as up to one minimum wage and more than one minimum wage;
  d) Skin color or race (White, Brown, Black, Asian, Indigenous), categorized as White and non-White.
- Presence of hypertension and diabetes
  e) Hypertension diagnosis was determined based on the question, "Has a doctor ever diagnosed you with arterial hypertension (high blood pressure)?", combined with the question, "Did the arterial hypertension (high blood pressure) occur only during a period of pregnancy?", and categorized as "no," "chronic hypertension," or "only during pregnancy."
  f) Diabetes diagnosis was determined based on the question "Has a doctor ever diagnosed you with diabetes?", combined with the question, "Did this diabetes occur only during a period of pregnancy?", and classified into the categories "yes" and "no" due to the small number of participants with diabetes.
- Adequacy of prenatal care
  g) Adequacy of prenatal care was defined based on a composite indicator that considered initiation of prenatal care during the first trimester of pregnancy and the completion of six or more prenatal visits. The indicator was classified as "yes" when both conditions were met and "no" when either condition was not met. The following questions were used to construct the indicator: "How far along in the pregnancy were you when you had your first prenatal visit?" and "How many prenatal visits did you have during this pregnancy?".
  h) Blood pressure measurement during prenatal care was assessed using the question, "During prenatal care, at how many visits was your blood pressure measured?", categorized as "yes" when blood pressure was measured at all visits and "no" otherwise;
  i) Receipt of guidance regarding the designated maternity hospital for delivery was assessed by the question, "During this prenatal care, were you informed/advised by the health service or health professional about the maternity hospital you should go to at the time of delivery?" and classified as "yes" or "no".
  j) The concept of "pilgrimage" for childbirth was constructed based on the questions, "Did the delivery take place at the health facility designated during prenatal care?" and "How many health services did you seek when you went into labor so that your child could be born?" The absence of pilgrimage was defined as cases in which there was no need to seek another health service for the delivery, whereas the presence of pilgrimage was defined as cases where it was necessary to seek one or more health services.
- Childbirth assistance
  k) Indicators related to childbirth care included delivery performed through the Brazilian Unified Health System (*Sistema Único de Saúde* – SUS), identified by the question "Was the delivery performed through the Unified Health System (SUS)?", and categorized as "yes" or "no;"
  l) Type of delivery was obtained from the question, "What was the type of delivery?", and classified as vaginal delivery or cesarean section;
  m) Delivery at the health facility designated during prenatal care was assessed using the question, "Was the delivery performed at the health facility designated during prenatal care?", and classified as "yes" or "no."
  n) Gestational age at birth was obtained from the question, "Number of weeks," and subsequently categorized as preterm (24 to 36 weeks), 37 to 38 weeks, 39 to 41 weeks, and 42 weeks or more.
  o) Labor was constructed using the questions, "What was the type of delivery?" and "Did you go into labor during this pregnancy?", and grouped into three categories: cesarean section without labor, cesarean section with labor, and vaginal delivery. Additionally, the primary reason for cesarean section was analyzed using the question, "What was the main reason for having a cesarean delivery?", with responses grouped into vaginal delivery, previous cesarean section, medical indication, and other.
- Problems in the child
  p) Problems related to the child were assessed using the question, "Did your child leave the hospital with you?", and classified as "death before hospital discharge," "hospital stay," or "other problem."
  q) Birth weight was obtained from the question, "Birth weight," and subsequently categorized as less than 2,500 g or 2,500 g or more. Information on whether the child was weighed at birth was obtained from the question, "Was your child weighed at birth?"
  r) Prematurity was determined based on the question, "How many weeks of pregnancy had elapsed at the time of delivery?", being classified as "less than 37 weeks" or "37 weeks or more."

### Statistical analysis

Proportional distributions were calculated for sociodemographic characteristics, prenatal care adequacy indicators, the presence of hypertension and diabetes, and indicators of childbirth care and newborn conditions, according to the occurrence of any childbirth complication.

To assess the statistical significance of the observed differences, Pearson's χ² tests were performed, with statistical significance set at p<5%.

To investigate factors associated with the occurrence of childbirth complications, bivariate logistic regression models were fitted, using the odds ratio (OR) and its respective 95% confidence intervals as measures of association. Associations between the outcome and sociodemographic variables, indicators of prenatal care adequacy, diagnoses of hypertension and diabetes mellitus, and factors related to childbirth care were evaluated.

Additionally, to analyze the consequences of childbirth complications in terms of problems affecting the baby, bivariate logistic regression models were fitted, using the OR as the measure of association.

All statistical analyses were performed using SPSS statistical software, version 21, with the Complex Samples module, taking into account the complex sampling design of the PNS.

#### Data availability statement:

The entire dataset supporting the results of this study is publicly available on the PNS website.

## RESULTS

The prevalence of any childbirth complication was 12.3% (95%CI 10.6–14.2), as shown in Table 1. Complications were more frequent among women with an educational level of incomplete high school or lower (14.4%) than among those with higher educational attainment (7.9%; p=0.031), and among those with a per capita household income of up to one minimum wage (13.9%) than among those with higher income (7.6%; p=0.002). Significantly higher prevalences were also observed among women with chronic hypertension (37.1%) and hypertension occurring only during pregnancy (29.8%) compared with those without hypertension (9.3%; p<0.001). In addition, differences were observed according to blood pressure measurement during prenatal care, with a prevalence of complications of 12.7% among women whose blood pressure was measured at every visit and 7.3% among the others (p=0.042).

**Table 1:** Comparison of sociodemographic variables, prenatal care adequacy indicators, and hypertension diagnosis according to the occurrence of any childbirth complication. National Health Survey, 2019.

Characteristic	Category	Childbirth complication						p-value
		Yes			No			
		n	%	95%CI	n	%	95%CI	
Total		353	12.3	(10.6–14.2)	2,522	87.7	(85.8–89.4)	-
Age group	15 to 24 years	97	10.4	(7.9–13.5)	839	89.6	(86.5–92.1)	0.242
	25 to 34 years	162	12.6	(10.3–15.4)	1,116	87.4	(84.6–89.7)	
	35 years or older	95	14.3	(10.7–19.0)	566	85.7	(81.1–89.3)	
Educational attainment	Incomplete high school	165	14.4	(11.6–17.8)	981	85.6	(82.2–88.4)	0.031
	Incomplete higher education	146	12.2	(10.0–14.8)	1,053	87.8	(85.2–90.0)	
	Completed higher education	42	7.9	(5.0–12.1)	488	92.1	(87.9–95.0)	
*Per capita* household income	Up to 1 MW	297	13.9	(11.9–16.2)	1,841	86.1	(83.8–88.1)	0.002
	More than 1 MW	56	7.6	(5.3–10.9)	681	92.4	(89.1–94.7)	
Race/skin color	White	100	10.0	(7.3–13.4)	904	90.0	(86.6–92.7)	0.076
	Non-White	253	13.5	(11.5–15.8)	1,618	86.5	(84.2–88.5)	
Adequate PN*	No	99	15.0	(11.5–19.4)	558	85.0	(80.6–88.5)	0.095
	Yes	254	11.5	(9.6–13.6)	1,963	88.5	(86.4–90.4)	
Guidance regarding the designated maternity hospital during PN	Yes	296	12.6	(10.7–14.9)	2,045	87.4	(85.1–89.3)	0.664
	No	52	11.7	(8.6–15.7)	396	88.3	(84.3–91.4)	
BP measured at all PN visits	No	15	7.3	(4.3–12.2)	191	92.7	(87.8–95.7)	0.042
	Yes	338	12.7	(10.9–14.7)	2,331	87.3	(85.3–89.1)	
Hypertension diagnosis	No	232	9.3	(7.9–10.9)	2,266	90.7	(89.1–92.1)	<0.001
	Yes	45	37.1	(24.4–51.9)	75	62.9	(48.1–75.6)	
	Only during pregnancy	76	29.8	(20.3–41.4)	180	70.2	(58.6–79.7)	

95%CI: 95% confidence interval; MW: minimum wage; PN: prenatal care; BP: blood pressure; p: p-value of the χ² test for comparison of proportions

* prenatal care initiated during the first trimester and six or more visits completed.

Childbirth care indicators were associated with the occurrence of childbirth complications (Table 2). Complications were more frequent among women who gave birth through SUS (14.5%; p=0.004), underwent cesarean section (13.9%; p=0.037), underwent cesarean section due to a medical indication or previous cesarean section (17.3%; p<0.001), and underwent cesarean section after the onset of labor (18.2%; p=0.006). In addition, the prevalence of complications was higher among preterm births (22.4%; p<0.001).

**Table 2:** Comparison of indicators related to childbirth care according to the occurrence of any childbirth complication. National Health Survey, 201.

Characteristic	Category	Childbirth complication						p-value
		Yes			No			
		n	%	95%CI	n	%	95%CI	
Delivery at the designated health facility	No	100	12.4	(9.8–15.7)	706	87.6	(84.3–90.2)	0.907
	Yes	253	12.2	(10.2–14.6)	1,815	87.8	(85.3–89.9)	
Delivery through SUS	No	61	7.1	(4.3–11.5)	795	92.9	(88.5–95.7)	0.004
	Yes	292	14.5	(12.5–16.7)	1,727	85.5	(83.3–87.5)	
Pilgrimage*	No	331	12.1	(10.3–14.1)	2,411	87.9	(85.9–89.7)	0.141
	Yes	22	16.6	(11.1–24.2)	111	83.4	(75.8–88.9)	
Type of delivery	Cesarean section	218	13.9	(11.4–16.8)	1,349	86.1	(83.2–88.6)	0.037
	Vaginal delivery	135	10.3	(8.3–12.7)	1,173	89.7	(87.3–91.7)	
Reason for cesarean section	Vaginal delivery	135	10.3	(8.3–12.7)	1,173	89.7	(87.3–91.7)	<0.001
	Previous CS or medical indication	173	17.3	(14.1–21.1)	828	82.7	(78.9–85.9)	
	Other	45	7.9	(4.8–12.8)	521	92.1	(87.2–95.2)	
Labor	CS without labor	119	11.6	(8.8–15.3)	903	88.4	(84.7–91.2)	0.006
	CS with labor	99	18.2	(13.9–23.5)	446	81.8	(76.5–86.1)	
	Vaginal delivery	135	10.3	(8.3–12.7)	1,173	89.7	(87.3–91.7)	
Gestational age (weeks)	Preterm (24 to 36)	84	22.4	(16.3–29.9)	292	77.6	(70.1–83.7)	<0.001
	Term (37 to 41)	220	10.4	(8.6–12.5)	1,904	89.6	(87.5–91.4)	
	Post-term (42 or more)	23	15.5	(9.7–23.9)	128	84.5	(76.1–90.3)	

95%CI: 95% confidence interval; SUS: Unified Health System (*Sistema Único de Saúde*); CS: cesarean section; p: p-value of the χ² test for comparison of proportions

* needed to seek care at more than one health service for delivery.

The results in Table 3 show that the occurrence of any childbirth complication was significantly higher among women who gave birth through SUS (14.5%), twice the prevalence observed among those who did not use SUS (7.1%). Women who underwent cesarean section had significantly higher odds of childbirth complications than those who had a vaginal delivery (OR=1.41; p=0.038), and the odds of having a cesarean section without entering labor were nearly twice as high (OR=1.93; p<0.001).

**Table 3:** Results of the bivariate logistic regression model with the occurrence of any childbirth complication as the outcome and sociodemographic characteristics, prenatal care adequacy, hypertension diagnosis, and childbirth care indicators as covariates. National Health Survey, 2019.

Characteristic	Category	OR	95%CI	p-value
Age group	35 years or older	1.26	(0.87–1.83)	0.215
	Less than 35 years	1.00	–	
Educational attainment	Incomplete high school	1.38	(1.01–1.89)	0.045
	Completed high school or higher	1.00	–	
Skin color	White	0.71	(0.49–1.04)	0.077
	Non-White	1.00	–	
Income	>1 MW	0.51	(0.33–0.79)	0.003
	≤1 MW	1.00	–	
Adequate prenatal care*	Yes	0.73	(0.51–1.06)	0.096
	No	1.00	–	
Type of delivery	Cesarean section	1.41	(1.02–1.92)	0.038
	Vaginal delivery	1.00	–	
Delivery through SUS	SUS	2.21	(1.28–3.82)	0.004
	Non-SUS	1.00	–	
Labor	Cesarean section without labor	1.93	(1.30–2.88)	0.001
	Cesarean section with labor	1.15	(0.78–1.68)	0.489
	Vaginal delivery	1.00	–	–
BP measured at all PN visits	No	1.83	(1.01–3.32)	0.045
	Yes	1.00	–	
Hypertension diagnosis	Yes	5.76	(3.08–10.77)	<0.001
	Only during pregnancy	4.14	(2.40–7.13)	<0.001
	No	1.00	–	–
Delivery at the health facility designated during PN	Yes	0.98	(0.70–1.38)	0.907
	No	1.00	–	
Pilgrimage**	Yes	1.45	(0.88–2.40)	0.143
	No	1.00	–	
Reason for cesarean section	Previous cesarean section	1.19	(0.60–2.34)	0.622
	Cesarean section due to a medical indication	1.97	(1.39–2.80)	<0.001
	Other reason	0.75	(0.42–1.33)	0.322
	No cesarean section	1.00	–	–

OR: odds ratio; 95%CI: 95% confidence interval; MW: minimum wage; SUS: Unified Health System (*Sistema Único de Saúde*); BP: blood pressure; PN: prenatal care; p: p-value of the statistical test

* prenatal care initiated during the first trimester and 6 or more visits completed;

** needed to seek care at more than one health service for delivery.

The multivariate model (Table 4) indicated that a diagnosis of hypertension (chronic or occurring only during pregnancy), delivery through SUS, cesarean section due to a medical indication or without labor, and gestational age <37 weeks were significantly associated with the outcome, even after adjustment for all other variables.

**Table 4:** Results of the multivariate logistic regression model with the occurrence of any childbirth complication as the outcome and sociodemographic factors, prenatal care indicators, hypertension diagnosis, and childbirth care indicators as covariates. National Health Survey, 2019.

Characteristic	OR	95%CI	p-value
Age ≥35	0.98	(0.65–1.46)	NS
Incomplete high school	0.98	(0.67–1.41)	NS
White skin color	0.82	(0.56–1.21)	NS
Income >1 MW	0.77	(0.47–1.26)	NS
Blood pressure measured at all prenatal care visits	2.16	(1.01–4.67)	0.049
Chronic hypertension	5.49	(2.90–10.41)	<0.001
Hypertension occurring only during pregnancy	4.18	(2.47–7.08)	<0.001
Cesarean section	1.35	(0.67–2.74)	NS
Delivery through SUS	1.99	(1.06–3.71)	0.031
Pilgrimage*	0.76	(0.39–1.50)	NS
Cesarean section due to previous cesarean section	1.24	(0.52–2.97)	NS
Cesarean section due to a medical indication	1.90	(1.06–3.41)	0.032
Cesarean section without labor	1.75	(1.10–2.80)	0.019
Gestational age <37 weeks	2.34	(1.62–3.38)	<0.001

OR: odds ratio; 95%CI: 95% confidence interval; NS: not significant; MW: minimum wage; SUS: Unified Health System (*Sistema Único de Saúde*); p: p-value of the statistical test

* needed to seek care at more than one health service for delivery.

The data in Table 5 show that, among mothers who experienced childbirth complications, the odds of the baby having a problem were 4.12 times higher (p<0.001), the odds of preterm birth (<37 weeks of gestation) were 2.43 times higher, and the odds of low birth weight (<2,500 g) were 2.59 times higher.

**Table 5:** Associations between the occurrence of problems affecting the baby and childbirth complications. National Health Survey, 2019.

Characteristic		Category	OR	95%CI	p-value
Outcome: Problems affecting the baby*					
	Childbirth complication	Yes	4.12	(2.48–6.85)	<0.001
		No	1.00	–	
Outcome: Prematurity (<37 weeks)					
	Childbirth complication	Yes	2.43	(1.60–3.68)	<0.001
		No	1.00	–	
Outcome: Low birth weight					
	Childbirth complication	Yes	2.59	(1.58–4.23)	<0.001
		No	1.00	–	

OR: odds ratio; 95%: 95% confidence interval; p: p-value of the test of the hypothesis OR=1

* problems affecting the baby – when the baby died, remained hospitalized, or had another problem (yes/no).

## DISCUSSION

Based on data from the 2019 PNS, it is evident that the occurrence of childbirth complications was associated with socioeconomic factors, maternal clinical conditions, and characteristics of childbirth care. The highest prevalences were observed among women with lower levels of education and income, those diagnosed with hypertension, and those who underwent cesarean section or experienced preterm birth. Recent studies reinforce that socioeconomic vulnerability, particularly low educational attainment and Black or Brown skin color, is a critical predictor of severe obstetric outcomes, with Black women exhibiting a significantly higher maternal mortality ratio than White women^21,22^.

Regarding childbirth care, the public health system predominantly serves pregnant women experiencing greater social vulnerability and has a significantly higher proportion of vaginal deliveries. The prevalence of any childbirth complication among women who gave birth through SUS was 14.5%, twice that observed in the private sector (7.1%). Higher frequencies of preterm birth (<37 weeks) and low birth weight were observed among women who gave birth through SUS.

However, data from the "Nascer no Brasil II" study indicate that, in the public sector, pregnant women are more likely to go into labor and have a vaginal delivery (54.4%) than those in the private sector, where elective interventions and cesarean sections without a clear medical indication predominate^5^. This coexistence of distinct care patterns reveals that, while the private sector faces excessive medicalization, SUS faces the challenge of managing cases of greater clinical complexity under structural constraints.

The analysis conducted in this study, stratified by mode of delivery, showed that the odds of experiencing any childbirth complication were 40% higher among women who underwent cesarean section than among those who had a vaginal delivery, particularly when the cesarean section was elective and performed before the onset of labor. The problems identified included iatrogenic prematurity, low birth weight, low Apgar scores, respiratory disorders, longer hospital stays, and greater need for neonatal intensive care, reinforcing that vaginal delivery, when clinically indicated, is associated with better neonatal outcomes^23^.

The excessive medicalization of childbirth in Brazil drove the cesarean section rate to 60% in 2024, far exceeding the 15% threshold recommended by the World Health Organization (WHO)^24,25^. Although essential in high-risk cases, the indiscriminate use of cesarean sections is associated with adverse outcomes and increased indirect maternal mortality^22^. This evidence underscores the urgent need to restrict inductions and elective surgeries for pregnancies at 39 weeks of gestation or later, aiming to protect the health of women and newborns^23,26^.

Hypertension emerged as the most important clinical factor associated with childbirth complications. Women with chronic hypertension or hypertension occurring only during pregnancy had 6-fold and 4-fold higher odds of complications, respectively. Recent national data confirm that hypertensive disorders account for nearly 20% of direct causes of maternal death in Brazil^27^. Eclampsia remains a significant cause of death, highlighting shortcomings in early management compared with developed countries^28-30^. Therefore, routine blood pressure measurement during prenatal care should be established as a low-cost, highly effective strategy for preserving maternal cardiovascular health^31^.

Maternal complications severely affect neonatal outcomes, particularly among women who give birth through SUS, where the in-hospital mortality rate was 17 per 1,000 pregnant women during the period analyzed. This situation is exacerbated by inequities in geographic access; an estimated 31% of pregnant women in Brazil need to travel to other municipalities to give birth, covering distances of up to 60 km^32^. This displacement disproportionately affects high-risk pregnant women, delaying care for critical complications and contributing to persistent infant morbidity and mortality^33,34^. The need for transfer to facilities with adequate neonatal intensive care remains a structural obstacle in Brazil, generating inequities in access to specialized care for preterm infants^35,36^.

In the United States, the choice of birth location is considered a factor strongly associated with maternal and infant mortality. To ensure the quality of care for pregnant women enrolled in Medicaid, the choice is made among in-network hospitals or hospitals with a neonatal intensive care unit (NICU), indicating the availability of advanced obstetric resources^35^.

Despite the complexity of changing childbirth care practices, the development of new strategies and the strengthening of initiatives in Brazil aimed at reducing cesarean section rates, strengthening obstetric resources within SUS, and improving the organization of transfers of pregnant women to public facilities with more adequate infrastructure could promote more effective care practices and reduce maternal and perinatal complications.

Among the limitations of the statistical analysis, a key issue concerns the variables used as "associated factors," as their occurrence may be concomitant with or subsequent to the outcome of interest. This weakens inferences regarding temporal ordering. Given that cesarean sections often result from obstetric complications, these variables may actually represent outcomes rather than predictors, suggesting the potential for reverse causation bias.

The limitations inherent to PNS must also be considered. Because the information is self-reported, the data are subject to recall bias, overreporting of socially desirable practices, difficulties in understanding the questions, and embarrassment when answering certain items. Additionally, the low frequency of reported diabetes may indicate underreporting resulting from a lack of awareness of the diagnosis, communication gaps between health professionals and users, or difficulties among respondents in recognizing the condition.

Limitations of the data collection instrument also affected the analysis of childbirth characteristics. Inconsistencies were observed in responses regarding the financing of delivery, including 89 women who reported giving birth in public maternity hospitals but indicated that their delivery was not covered by SUS. Consequently, the "SUS delivery" variable was analyzed according to the original categories (yes/no), considering that most negative responses corresponded to deliveries performed in the private sector. Furthermore, analysis of the place of delivery was precluded by the structure of the response categories, which were heterogeneous and did not allow for a clear distinction between public and private care. This limitation hindered consistent analytical grouping and may have contributed to misclassification, highlighting the need for more concise and standardized response categories in future research.
