# Supplementary material for: Childbirth complications in Brazil: An analysis of socioeconomic, epidemiological, and health care factors
Source: Rev Bras Epidemiol. 2026 Oct 19;29:e260053. doi: 10.1590/1980-549720260053 (PMC13679880; doi:10.1590/1980-549720260053)
Supplement: Translation

# Translation: Complicações no parto no Brasil: análise de fatores socioeconômicos, epidemiológicos e assistenciais

**DOI:** 10.1590/1980-549720260053.2

**Authors:** Narayani Martins Rocha, Wanessa da Silva de Almeida, Célia Landmann Szwarcwald

**Affiliations:** I Fundação Oswaldo Cruz, Instituto de Comunicação e Informação Científica e Tecnológica em Saúde – Rio de Janeiro (RJ), Brasil.; II Fundação Oswaldo Cruz, Instituto Nacional de Infectologia Evandro Chagas – Rio de Janeiro (RJ), Brasil.

**Keywords:** Parto, Parto normal, Cesárea, Complicações do Trabalho de Parto, Sistema Único de Saúde, Brasil

## Abstract

**Objetivo:**

Analisar os fatores associados à ocorrência de complicações no parto no Brasil.

**Métodos:**

Estudo transversal com informações da Pesquisa Nacional de Saúde de 2019. Foram analisados dados de 2.875 mulheres. Para investigar a associação entre fatores sociodemográficos e indicadores de assistência com a ocorrência de complicações no parto (desfecho), utilizaram-se testes qui-quadrado e regressão logística.

**Resultados:**

A prevalência de complicações no parto foi de 12,3% (IC95%10,6–14,2). Complicações no parto foram mais prevalentes entre mulheres com menor escolaridade (14,4%) e renda (13,9%), hipertensão crônica (37,1%) ou gestacional (29,8%), parto cesáreo (13,9%) e parto pelo Sistema Único de Saúde – SUS (14,5%), comparadas aos respectivos grupos de referência (p<0,05). O modelo multivariado indicou associação significativa do desfecho com hipertensão crônica (OR=5,49; IC95% 2,90–10,41) e gestacional (OR=4,18; IC95% 2,47–7,08), ausência de aferição da pressão arterial nas consultas (OR=2,16; IC95% 1,01–4,67), parto pelo SUS (OR=1,99; IC95% 1,06–3,71), e cesariana sem trabalho de parto (OR=1,75; IC95% 1,10–2,80) ou por indicação médica (OR=1,90; IC95% 1,06–3,41). Entre mulheres com complicação no parto, houve maior chance de intercorrências neonatais (OR=4,12; IC95% 2,48–6,85), baixo peso ao nascer (OR=2,59; IC95% 1,58–4,23) e prematuridade (OR=2,43; IC95% 1,60–3,68).

**Conclusão:**

Fatores clínicos e características da assistência ao parto estiveram associados à ocorrência de complicações no parto no Brasil. Além disso, mulheres com complicações no parto tiveram maior chance de desfechos neonatais adversos.

## INTRODUÇÃO

O parto é um evento relevante no ciclo reprodutivo e importante indicador da qualidade da atenção materno-infantil. Apesar dos avanços nas últimas décadas quanto a cobertura e acesso aos serviços de saúde, persistem fragilidades na assistência obstétrica, refletidas na ocorrência de complicações maternas e neonatais evitáveis^1-4^.

As desigualdades regionais e socioeconômicas agravam esse cenário. Mulheres de baixa renda e residentes nas regiões Norte e Nordeste enfrentam maior dificuldade de acesso a serviços de qualidade, sendo mais vulneráveis a atrasos no atendimento, peregrinação por vaga hospitalar e partos em condições precárias^5,6^. Essas disparidades impactam diretamente a morbimortalidade materna e neonatal^7^, ainda acima das metas internacionais de desenvolvimento sustentável.

Embora políticas públicas como a Rede Cegonha e a Política Nacional de Humanização tenham buscado humanizar o cuidado e qualificar a assistência ao parto com a redução de intervenções desnecessárias^8-10^, a resistência institucional, barreiras estruturais e de recursos humanos limitam a efetivação dessas diretrizes^11^.

O foco central desse problema reside nas complicações maternas durante o parto ou no pós-parto e permanecem como as principais causas de mortalidade materna no país, frequentemente decorrentes da demora no diagnóstico e no manejo adequado das intercorrências^12^. A ocorrência desses desfechos negativos é potencializada pelo uso indiscriminado de intervenções, como uso de ocitocina, episiotomia e realização de cesarianas desnecessárias, que elevam os riscos obstétricos de curto e longo prazos^13,14^.

Do ponto de vista neonatal, problemas no bebê podem estar relacionados às condições do parto e ao tipo de assistência recebida. As complicações mais comuns decorrem da demora na extração fetal ou da falta de monitoramento adequado do bem-estar fetal^15^. A prematuridade, muitas vezes associada a cesarianas eletivas antes de 39 semanas, aumenta a vulnerabilidade do recém-nascido a infecções e complicações respiratórias^16^. Esse quadro pode ser agravado pela ausência de infraestrutura e de profissionais capacitados para reanimação neonatal, o que contribui para eventos adversos evitáveis^17,18^.

Diante do exposto, o presente estudo objetiva analisar os fatores associados à ocorrência de complicações no parto, utilizando dados da Pesquisa Nacional de Saúde (2019). Foram considerados fatores sociodemográficos: indicadores de adequação da assistência pré-natal e ao parto, presença de hipertensão arterial e suas associações com problemas maternos e perinatais.

## MÉTODOS

### Fonte de dados

Este estudo utilizou dados da Pesquisa Nacional de Saúde (PNS), edição de 2019, referente aos dados do Módulo S – Atendimento ao PN, que reúne informações referentes ao acompanhamento da gestação e à assistência ao parto no último nascimento.

A PNS é uma pesquisa domiciliar de abrangência nacional conduzida pelo Instituto Brasileiro de Geografia e Estatística (IBGE) em parceria com o Ministério da Saúde. A PNS faz parte do Sistema Integrado de Pesquisas Domiciliares (SIPD) do IBGE. Seu principal objetivo é produzir informações representativas sobre o perfil de saúde da população brasileira, suas condições de saúde e fatores de risco, além de subsidiar a avaliação do desempenho do sistema de saúde^19^.

A PNS-2019 foi aprovada pela Comissão Nacional de Ética em Pesquisa (CONEP) em agosto de 2019 (parecer n. 3.529.376).

### Plano amostral e amostra

A amostra da PNS foi selecionada como uma subamostra da Amostra Mestra do IBGE, realizada em três estágios de seleção. No primeiro estágio, as Unidades Primárias de Amostragem (UPA) são selecionadas por amostragem aleatória simples, considerando a estratificação da Amostra Mestra. No segundo estágio, em cada UPA são selecionados os domicílios por amostragem aleatória simples. No terceiro estágio, um morador do domicílio é escolhido aleatoriamente para responder ao questionário individual^20^.

A amostra é composta de mulheres de 15 anos ou mais que tiveram pelo menos um parto nos dois anos anteriores à realização da pesquisa, ou seja, entre 28 de julho de 2017 e 27 de julho de 2019.

### Variáveis do estudo

- Desfecho: Teve alguma complicação no parto, medida pela questão "Teve alguma complicação durante o parto?", que foi autorreferida pelas participantes.
- Variáveis sociodemográficas
  a) Faixa etária materna, obtida a partir da pergunta "Idade do morador na data de referência", posteriormente categorizada em 15 a 24 anos, 25 a 34 anos e 35 anos ou mais;
  b) Grau de escolaridade, obtido pela pergunta "Nível de instrução mais elevado alcançado", cujas categorias foram agrupadas em ensino médio incompleto, superior incompleto e superior completo;
  c) Renda domiciliar *per capita*, derivada da pergunta "Faixa de rendimento domiciliar *per capita*", categorizada em até um salário-mínimo e maior que um salário-mínimo;
  d) Cor da pele ou raça (branca, parda, negra, amarela, indígena), sendo consideradas as categorias branca e não branca.
- Presença de hipertensão e diabetes
  e) O diagnóstico de hipertensão foi obtido a partir da pergunta "Algum médico já lhe deu o diagnóstico de hipertensão arterial (pressão alta)?", combinada com a pergunta "A hipertensão arterial (pressão alta) ocorreu apenas durante algum período de gravidez?", sendo categorizada em "não", "hipertensão arterial crônica" e "somente durante a gravidez".
  f) O diagnóstico de diabetes foi obtido a partir da pergunta "Algum médico já lhe deu o diagnóstico de diabetes?", combinada com a pergunta "Esse diabetes ocorreu apenas durante algum período de gravidez?", sendo classificada apenas nas categorias "sim" e "não" devido ao número pequeno de participantes com diabetes.
- Adequação do pré-natal
  g) A adequação do pré-natal foi definida a partir de um indicador composto que considerou o início do acompanhamento no primeiro trimestre gestacional e a realização de seis ou mais consultas de pré-natal, sendo classificado como "sim" quando ocorrem as duas condições e "não" quando uma delas não ocorre. Para sua construção, foram utilizadas as perguntas "Quanto tempo de gravidez tinha quando fez a primeira consulta pré-natal?" e "Quantas consultas de pré-natal fez durante esta gravidez?".
  h) A aferição da pressão arterial durante o pré-natal foi feita por meio da pergunta "Durante o pré-natal, em quantas consultas mediram sua pressão arterial?", categorizada em todas as consultas como "sim" ou "não", caso contrário;
  i) Recebimento de orientação sobre a maternidade de referência para o parto, avaliado pela pergunta "Durante este pré-natal você foi informada/orientada pelo serviço de saúde/profissional de saúde sobre a maternidade para qual deveria se dirigir no momento do parto?", classificada em "sim" e "não".
  j) A peregrinação para realização do parto foi construída a partir das perguntas "O parto foi realizado no estabelecimento de saúde indicado no pré-natal?" e "Quantos serviços de saúde procurou quando entrou em trabalho de parto para que seu(sua) filho(a) pudesse nascer?". Considerou-se ausência de peregrinação quando não houve necessidade de procurar outro serviço para a realização do parto e presença de peregrinação quando foi necessário procurar um ou mais serviços de saúde.
- Assistência ao parto
  k) Os indicadores relacionados à assistência ao parto incluíram a realização do parto pelo Sistema Único de Saúde (SUS), identificada pela pergunta "O parto foi feito através do Sistema Único de Saúde (SUS)?", categorizada em "sim" e "não";
  l) O tipo de parto, obtido pela pergunta "Qual foi o tipo de parto?", classificado em parto normal ou cesariana;
  m) Realização do parto no estabelecimento de saúde indicado durante o pré-natal, avaliada pela pergunta "O parto foi realizado no estabelecimento de saúde indicado no pré-natal?", classificada em "sim" e "não".
  n) A idade gestacional ao nascimento foi obtida pela pergunta "Quantidade de semanas", sendo posteriormente categorizada em pré-termo (24 a 36 semanas, 37 a 38 semanas, 39 a 41 semanas e 42 semanas ou mais.
  o) A variável trabalho de parto foi construída utilizando as perguntas "Qual foi o tipo de parto?" e "Nesta gravidez, entrou em trabalho de parto?", sendo agrupada em três categorias: cesariana sem trabalho de parto, cesariana com trabalho de parto e parto normal. Complementarmente, foi analisado o principal motivo da cesariana por meio da pergunta "Qual o principal motivo de ter tido parto cesáreo?", cujas categorias foram agrupadas em parto normal, cesariana anterior, indicação médica e outro.
- Problemas na criança
  p) Os problemas relacionados à criança foram avaliados a partir da pergunta "Seu(sua) filho(a) saiu do hospital junto com você?", sendo classificados em "óbito antes da alta hospitalar", "permanência hospitalar" ou "outro problema".
  q) O peso ao nascer foi obtido pela pergunta "Peso ao nascer", posteriormente categorizado em menor que 2.500 g e igual ou superior a 2.500 g. A informação sobre pesagem ao nascer foi obtida pela pergunta "Seu(sua) filho(a) foi pesado ao nascer?".
  r) A prematuridade foi determinada a partir da pergunta "Quantas semanas de gravidez tinha no momento do parto?", sendo classificada em "menos de 37 semanas" e "37 semanas ou mais".

### Análise estatística

Foram calculadas as distribuições proporcionais das características sociodemográficas, os indicadores de adequação do pré-natal, a presença de hipertensão e diabetes, e os indicadores de assistência ao parto e das condições do recém-nascido, segundo a ocorrência de alguma complicação no parto.

Para verificar a significância estatística das diferenças encontradas, foram realizados testes χ² de Pearson, estabelecendo o nível descritivo do teste menor do que 5% como significativo (valor de p<5%).

Para investigar os fatores associados à ocorrência de complicação no parto, foram ajustados modelos de regressão logística bivariada, utilizando-se a razão de chances (odds ratio – OR) e seus respectivos intervalos de confiança de 95% como medidas de associação. Foram avaliadas as associações entre o desfecho e as variáveis sociodemográficas, os indicadores de adequação do pré-natal, o diagnóstico de hipertensão arterial e diabetes mellitus e os fatores relacionados à assistência ao parto.

Para analisar a associação das variáveis à ocorrência de complicação no parto, foram ajustados modelos de regressão logística multivariada. As associações entre o desfecho e cada variável independente do estudo foram analisadas, controlando-se por todas as demais variáveis, utilizando-se a razão de chances (OR) como medida de associação.

Adicionalmente, para a análise das consequências da ocorrência de alguma complicação no parto em problemas no bebê, foram realizados modelos de regressão logística bivariada, utilizando-se o OR como medida de associação.

Todas as análises estatísticas foram realizadas no *software* estatístico SPSS, versão 21, utilizando o módulo Complex Samples, considerando o desenho complexo de amostragem da PNS.

#### Declaração de disponibilidade de dados:

Todo o conjunto de dados que dá suporte aos resultados deste estudo é público, disponível no site da Pesquisa Nacional de Saúde (PNS).

## RESULTADOS

A prevalência de alguma complicação no parto foi de 12,3% (IC95% 10,6–14,2), conforme Tabela 1. As complicações foram mais frequentes entre mulheres com escolaridade até o ensino médio incompleto (14,4%) em comparação às de maior escolaridade (7,9%; p=0,031) e entre aquelas com renda domiciliar *per capita* de até um salário-mínimo (13,9%) em relação às de maior renda (7,6%; p=0,002). Também foram observadas prevalências significativamente mais elevadas entre mulheres com hipertensão arterial (37,1%) e hipertensão apenas durante a gestação (29,8%), quando comparadas às sem hipertensão (9,3%; p<0,001). Além disso, houve diferença segundo a aferição da pressão arterial durante o pré-natal, com prevalência de complicação de 12,7% entre as mulheres que tiveram a pressão aferida em todas as consultas e de 7,3% entre as demais (p=0,042).

**Tabela 1:** Comparação de variáveis sociodemográficas, indicadores de adequação do pré-natal e presença de diagnóstico de hipertensão arterial segundo a ocorrência de alguma complicação no parto. Pesquisa Nacional de Saúde, 2019.

Variável	Categoria	Complicação no parto						Valor p
		Sim			Não			
		n	%	IC95%	n	%	IC95%	
Total		353	12,3	(10,6–14,2)	2.522	87,7	(85,8–89,4)	-
Faixa etária	15 a 24 anos	97	10,4	(7,9–13,5)	839	89,6	(86,5–92,1)	0,242
	25 a 34 anos	162	12,6	(10,3–15,4)	1.116	87,4	(84,6–89,7)	
	35 anos ou mais	95	14,3	(10,7–19,0)	566	85,7	(81,1–89,3)	
Grau de escolaridade	Médio incompleto	165	14,4	(11,6–17,8)	981	85,6	(82,2–88,4)	0,031
	Superior incompleto	146	12,2	(10,0–14,8)	1.053	87,8	(85,2–90,0)	
	Superior completo	42	7,9	(5,0–12,1)	488	92,1	(87,9–95,0)	
Renda domiciliar *per capita*	Até 1 SM	297	13,9	(11,9–16,2)	1.841	86,1	(83,8–88,1)	0,002
	Maior que 1 SM	56	7,6	(5,3–10,9)	681	92,4	(89,1–94,7)	
Raça/cor	Branca	100	10,0	(7,3–13,4)	904	90,0	(86,6–92,7)	0,076
	Não branca	253	13,5	(11,5–15,8)	1.618	86,5	(84,2–88,5)	
PN adequado*	Não	99	15,0	(11,5–19,4)	558	85,0	(80,6–88,5)	0,095
	Sim	254	11,5	(9,6–13,6)	1.963	88,5	(86,4–90,4)	
Orientação sobre a maternidade no PN	Sim	296	12,6	(10,7–14,9)	2.045	87,4	(85,1–89,3)	0,664
	Não	52	11,7	(8,6–15,7)	396	88,3	(84,3–91,4)	
PA medida em todas as consultas de PN	Não	15	7,3	(4,3–12,2)	191	92,7	(87,8–95,7)	0,042
	Sim	338	12,7	(10,9–14,7)	2.331	87,3	(85,3–89,1)	
Diagnóstico de hipertensão arterial	Não	232	9,3	(7,9–10,9)	2.266	90,7	(89,1–92,1)	<0,001
	Sim	45	37,1	(24,4–51,9)	75	62,9	(48,1–75,6)	
	Somente durante a gravidez	76	29,8	(20,3–41,4)	180	70,2	(58,6–79,7)	

IC95%: intervalo de confiança de 95%; SM: salário mínimo; PN: pré-natal; PA: pressão arterial; p: valor do nível descritivo do teste χ² de comparação de proporções

* pré-natal com início no primeiro trimestre e 6 ou mais consultas realizadas.

Os indicadores de assistência ao parto apresentaram associação com a ocorrência de complicação no parto (Tabela 2). As complicações foram mais frequentes entre mulheres que realizaram o parto pelo SUS (14,5%; p=0,004), tiveram parto cesáreo (13,9%; p=0,037), realizaram cesariana por indicação médica ou cesariana anterior (17,3%; p<0,001) e foram submetidas à cesariana após entrada em trabalho de parto (18,2%; p=0,006). Além disso, a prevalência de complicação foi maior entre os partos pré-termo (22,4%; p<0,001).

**Tabela 2:** Comparação de indicadores relacionados à assistência ao parto segundo a ocorrência de alguma complicação no parto. Pesquisa Nacional de Saúde, 2019.

Variável	Categoria	Complicação no parto						Valor p
		Sim			Não			
		n	%	IC95%	n	%	IC95%	
Parto realizado no serviço indicado	Não	100	12,4	(9,8–15,7)	706	87,6	(84,3–90,2)	0,907
	Sim	253	12,2	(10,2–14,6)	1.815	87,8	(85,3–89,9)	
Parto realizado no SUS	Não	61	7,1	(4,3–11,5)	795	92,9	(88,5–95,7)	0,004
	Sim	292	14,5	(12,5–16,7)	1.727	85,5	(83,3–87,5)	
Peregrinação*	Não	331	12,1	(10,3–14,1)	2.411	87,9	(85,9–89,7)	0,141
	Sim	22	16,6	(11,1–24,2)	111	83,4	(75,8–88,9)	
Tipo de parto	Cesariana	218	13,9	(11,4–16,8)	1.349	86,1	(83,2–88,6)	0,037
	Normal	135	10,3	(8,3–12,7)	1.173	89,7	(87,3–91,7)	
Motivo da cesariana	Parto normal	135	10,3	(8,3–12,7)	1.173	89,7	(87,3–91,7)	<0,001
	CS anterior ou indicação médica	173	17,3	(14,1–21,1)	828	82,7	(78,9–85,9)	
	Outro	45	7,9	(4,8–12,8)	521	92,1	(87,2–95,2)	
Trabalho de parto	CS sem trabalho de parto	119	11,6	(8,8–15,3)	903	88,4	(84,7–91,2)	0,006
	CS com trabalho de parto	99	18,2	(13,9–23,5)	446	81,8	(76,5–86,1)	
	Parto normal	135	10,3	(8,3–12,7)	1.173	89,7	(87,3–91,7)	
Idade gestacional (semanas)	Pré-termo (24 a 36)	84	22,4	(16,3–29,9)	292	77,6	(70,1–83,7)	<0,001
	A termo (37 a 41)	220	10,4	(8,6–12,5)	1.904	89,6	(87,5–91,4)	
	Pós-termo (42 ou mais)	23	15,5	(9,7–23,9)	128	84,5	(76,1–90,3)	

IC95%: intervalo de confiança de 95%; SUS: Sistema Único de Saúde; CS: cesariana; p: valor do nível descritivo do teste χ² de comparação de proporções

* precisou procurar mais de um serviço de saúde para a realização do parto.

Os resultados da Tabela 3 mostram que a ocorrência de alguma complicação no parto foi significativamente maior entre as mulheres que tiveram parto pelo SUS (14,5%) – o dobro das parturientes que não usaram o SUS (7,1%). Mulheres que fizeram cesariana tiveram chance significativamente maior do que as que tiveram parto normal (OR=1,41; p=0,038), e a chance de ter feito cesariana sem entrar em trabalho de parto foi quase 2 vezes maior (OR=1,93; p<0,001).

**Tabela 3:** Resultados do modelo bivariado de regressão logística tendo como desfecho a ocorrência de alguma complicação no parto e como covariáveis as características sociodemográficas, a adequação do pré-natal, o diagnóstico de hipertensão e os indicadores de assistência ao parto. Pesquisa Nacional de Saúde, 2019.

Variável	Categoria	OR	IC95%	Valor p
Faixa etária	35 anos ou mais	1,26	(0,87–1,83)	0,215
	Menos de 35 anos	1,00	–	
Grau de escolaridade	Médio incompleto	1,38	(1,01–1,89)	0,045
	Médio completo ou mais	1,00	–	
Cor da pele	Branca	0,71	(0,49–1,04)	0,077
	Não branca	1,00	–	
Renda	>1 SM	0,51	(0,33–0,79)	0,003
	≤1 SM	1,00	–	
Pré-natal adequado*	Sim	0,73	(0,51–1,06)	0,096
	Não	1,00	–	
Tipo de parto	Cesariana	1,41	(1,02–1,92)	0,038
	Normal	1,00	–	
Parto pelo SUS	SUS	2,21	(1,28–3,82)	0,004
	Não SUS	1,00	–	
Trabalho de parto	Cesariana sem trabalho de parto	1,93	(1,30–2,88)	0,001
	Cesariana com trabalho de parto	1,15	(0,78–1,68)	0,489
	Parto normal	1,00	–	–
PA medida em todas as consultas de PN	Não	1,83	(1,01–3,32)	0,045
	Sim	1,00	–	
Diagnóstico de hipertensão arterial	Sim	5,76	(3,08–10,77)	<0,001
	Apenas durante a gravidez	4,14	(2,40–7,13)	<0,001
	Não	1,00	–	–
Parto realizado no serviço indicado no pré-natal	Sim	0,98	(0,70–1,38)	0,907
	Não	1,00	–	
Peregrinação**	Sim	1,45	(0,88–2,40)	0,143
	Não	1,00	–	
Motivo da cesariana	Cesariana anterior	1,19	(0,60–2,34)	0,622
	Cesariana por indicação médica	1,97	(1,39–2,80)	<0,001
	Outro motivo	0,75	(0,42–1,33)	0,322
	Não realizou cesariana	1,00	–	–

OR: odds ratio; IC95%: intervalo de confiança de 95%; SM: salário mínimo; SUS: Sistema Único de Saúde; PA: pressão arterial; PN: pré-natal; p: valor do nível descritivo do teste

* pré-natal com início no primeiro trimestre e 6 ou mais consultas realizadas;

** precisou procurar mais de um serviço de saúde para a realização do parto.

O modelo multivariado (Tabela 4) indicou que ter diagnóstico de hipertensão (crônica ou gestacional), parto pelo SUS, ter feito cesariana por indicação médica ou cesariana sem trabalho de parto e idade gestacional <37 semanas foram as variáveis significativamente associadas ao desfecho, mesmo após o controle de todas as demais.

**Tabela 4:** Resultados do modelo multivariado de regressão logística tendo como desfecho a ocorrência de alguma complicação no parto e como covariáveis os fatores sociodemográficos, os indicadores da atenção pré-natal, o diagnóstico de hipertensão e os indicadores de assistência ao parto. Pesquisa Nacional de Saúde, 2019.

Variável	OR	IC95%	Valor p
Idade ≥35	0,98	(0,65–1,46)	NS
Ensino médio incompleto	0,98	(0,67–1,41)	NS
Cor da pele branca	0,82	(0,56–1,21)	NS
Renda >1 SM	0,77	(0,47–1,26)	NS
Pressão arterial medida em todas as consultas	2,16	(1,01–4,67)	0,049
Hipertensão crônica	5,49	(2,90–10,41)	<0,001
Hipertensão somente na gravidez	4,18	(2,47–7,08)	<0,001
Parto cesariana	1,35	(0,67–2,74)	NS
Parto pelo SUS	1,99	(1,06–3,71)	0,031
Peregrinação*	0,76	(0,39–1,50)	NS
Parto cesariana pelo motivo de ter feito cesariana anterior	1,24	(0,52–2,97)	NS
Fez cesariana por indicação médica	1,90	(1,06–3,41)	0,032
Fez cesariana sem entrar em trabalho de parto	1,75	(1,10–2,80)	0,019
Idade gestacional <37 semanas	2,34	(1,62–3,38)	<0,001

OR: odds ratio (razão dos produtos cruzados); IC95%: intervalo de confiança de 95%; NS: não significativo; SM: salário mínimo; SUS: Sistema Único de Saúde; p: valor do nível descritivo do teste

* precisou procurar mais de um serviço de saúde para a realização do parto.

Os dados da Tabela 5 mostram que, entre as mães que tiveram complicação no parto, a chance de ter tido um problema no bebê foi 4,12 vezes maior (p<0,001), 2,43 vezes maior de bebês prematuros (<37 semanas de gestação) e 2,59 vezes maior de baixo peso ao nascer (<2500 g).

**Tabela 5:** Associações da ocorrência de problemas no bebê em decorrência de alguma complicação no parto. Pesquisa Nacional de Saúde, 2019.

Variável		Categoria	OR	IC95%	Valor p
Desfecho: Problemas no bebê*					
	Complicação no parto	Sim	4,12	(2,48–6,85)	<0,001
		Não	1,00	–	
Desfecho: Prematuridade (<37 semanas)					
	Complicação no parto	Sim	2,43	(1,60–3,68)	<0,001
		Não	1,00	–	
Desfecho: Baixo peso ao nascer					
	Complicação no parto	Sim	2,59	(1,58–4,23)	<0,001
		Não	1,00	–	

OR: odds ratio; IC95%: intervalo de confiança de 95%; p: valor do nível descritivo do teste da hipótese de OR=1

* problemas no bebê – quando o bebê faleceu, permaneceu internado ou teve outro problema (sim/não).

## DISCUSSÃO

Com base nos dados da PNS 2019, pode-se verificar que a ocorrência de complicações no parto esteve associada a fatores socioeconômicos, condições clínicas maternas e características da assistência ao parto. As maiores prevalências foram observadas entre mulheres com menor escolaridade e renda, com diagnóstico de hipertensão arterial, submetidas a parto cesáreo e com parto pré-termo. Estudos recentes reforçam que a vulnerabilidade socioeconômica, especialmente a baixa escolaridade e a raça/cor preta ou parda, são preditores críticos de desfechos obstétricos graves, com mulheres negras apresentando uma razão de mortalidade materna significativamente superior à de mulheres brancas^21,22^.

Quanto à assistência ao parto, no sistema público prevalece o atendimento de gestantes em maior vulnerabilidade social e proporção significativamente maior do parto normal. A prevalência de alguma complicação no parto entre mulheres que realizaram o parto pelo SUS foi de 14,5%, 2 vezes maior do que a encontrada no setor privado (7,1%). No SUS, observaram-se maiores frequências de prematuridade (<37 semanas) e baixo peso ao nascer.

Entretanto, dados da pesquisa "Nascer no Brasil II" indicam que, no setor público, há uma maior probabilidade de a gestante entrar em trabalho de parto e ter acesso ao parto vaginal (54,4%) em comparação ao setor privado, onde prevalecem intervenções eletivas e cesarianas sem indicação médica clara^5^. Essa coexistência de padrões assistenciais distintos revela que, enquanto o setor privado sofre com a medicalização excessiva, o SUS enfrenta o desafio de manejar casos de maior complexidade clínica sob restrições estruturais.

Pela análise, realizada neste estudo, segundo o tipo de parto, foi possível constatar que a chance de ocorrência de alguma complicação no parto é 40% maior para o parto cesariana em comparação ao parto vaginal, especialmente quando a cesariana é eletiva e realizada sem que a gestante entre em trabalho de parto. Entre os problemas, destacam-se prematuridade iatrogênica, baixo peso ao nascer, Apgar reduzido, distúrbios respiratórios e maior tempo de internação e de necessidade de UTI neonatal, reforçando que o parto vaginal, quando clinicamente indicado, favorece melhores desfechos neonatais^23^.

A medicalização excessiva do parto no Brasil elevou a taxa de cesarianas para 60% em 2024, superando drasticamente o limite de 15% recomendado pela OMS^24,25^. Embora essencial em casos de risco, seu uso indiscriminado está associado a desfechos adversos e ao aumento da mortalidade materna indireta^22^. Tais evidências reforçam a urgência de restringir induções e cirurgias eletivas para gestações a partir de 39 semanas, visando proteger a saúde de mulheres e neonatos^23,26^.

A hipertensão arterial emergiu como o fator clínico mais determinante para complicações no parto. Mulheres com hipertensão crônica ou gestacional apresentaram chances de complicações 6 e 4 vezes maiores, respectivamente. Dados nacionais recentes confirmam que os transtornos hipertensivos respondem por quase 20% das causas diretas de óbito materno no Brasil^27^. A eclâmpsia continua sendo uma causa relevante de morte, evidenciando falhas no manejo precoce em comparação a países desenvolvidos^28-30^. Portanto, a aferição rotineira da pressão arterial no pré-natal deve ser consolidada como uma estratégia de baixo custo e alta eficácia para a preservação da saúde cardiovascular materna^31^.

As complicações maternas impactam severamente os desfechos neonatais, especialmente no SUS, onde a taxa de mortalidade hospitalar foi de 17 por 1.000 gestantes no período analisado. Esse cenário é agravado por iniquidades no acesso geográfico; estima-se que 31% das gestantes brasileiras precisem viajar para outros municípios para dar à luz, percorrendo distâncias que podem chegar a 60 km^32^. Esse deslocamento afeta desproporcionalmente gestantes de alto risco, retardando o atendimento a intercorrências críticas e contribuindo para a manutenção da morbimortalidade infantil^33,34^. A necessidade de transferência para serviços com UTI neonatal adequada ainda é um obstáculo estrutural no Brasil, gerando iniquidades no acesso aos cuidados especializados para prematuros^35,36^.

Nos Estados Unidos da América, a escolha do local de nascimento é considerada um fator fortemente associado à mortalidade materna e infantil. Para garantir a qualidade dos cuidados das gestantes inscritas no Medicaid, a seleção é realizada entre hospitais da rede ou hospitais com Unidade de Terapia Intensiva (UTI) neonatal, que indicam recursos obstétricos avançados^35^.

Apesar da complexidade em mudar as práticas de assistência ao parto, o desenvolvimento de novas estratégias e o fortalecimento de iniciativas no Brasil voltadas à redução das cesarianas, a impulsionar os recursos obstétricos no SUS e a melhorar a organização da transferência de gestantes para serviços públicos com estrutura mais adequada poderão promover práticas assistenciais mais eficazes, reduzindo as complicações maternas e perinatais.

Entre as limitações da análise estatística desse estudo, destacam-se as variáveis utilizadas como "fatores associados", cuja ocorrência pode ser concomitante ou posterior ao desfecho de interesse. Isso fragiliza a inferência de temporalidade. Considerando que cesarianas muitas vezes decorrem de intercorrências obstétricas, tais variáveis podem configurar desfechos em vez de preditores, sugerindo potencial viés de causalidade reversa.

Também devem ser consideradas as limitações inerentes à Pesquisa Nacional de Saúde (PNS). Por se tratar de informações autorreferidas, os dados estão sujeitos a viés de memória, superestimação de práticas socialmente desejáveis, dificuldades de compreensão das perguntas e constrangimento ao responder determinados itens. Adicionalmente, a baixa frequência de relatos de diabetes pode indicar subnotificação decorrente do desconhecimento do diagnóstico, falhas na comunicação entre profissionais e usuárias ou dificuldades de reconhecimento da condição pelas entrevistadas.

Limitações do instrumento de coleta também afetaram a análise das características do parto. Foram observadas inconsistências nas respostas sobre o financiamento do parto, a exemplo de 89 mulheres que declararam ter realizado o parto em maternidades públicas, mas informaram que o parto não ocorreu pelo SUS. Diante disso, a variável "parto pelo SUS" foi analisada conforme as categorias originais (sim/não), considerando que a maioria das respostas negativas correspondia a partos realizados no setor privado. Além disso, a análise do local de realização do parto foi inviabilizada pela estrutura das categorias de resposta, que eram heterogêneas e não permitiam distinguir claramente atendimentos públicos e privados. Essa limitação dificultou agrupamentos analíticos consistentes e pode ter contribuído para erros de classificação, indicando a necessidade de categorias mais sintéticas e padronizadas em futuras pesquisas.
